# A Serious Game to Study Reduced Field of View in Keyhole Surgery: Development and Experimental Study

**DOI:** 10.2196/56269

**Published:** 2025-02-11

**Authors:** Phoebe Whitley, Connor Creasey, Matthew J Clarkson, Stephen Thompson

**Affiliations:** 1 Department of Medical Physics and Biomedical Engineering Faculty of Engineering Sciences University College London London United Kingdom; 2 UCL Hawkes Institute Faculty of Engineering Sciences University College London London United Kingdom; 3 Advanced Research Computing University College London London United Kingdom

**Keywords:** keyhole surgery, laparoscopic surgery, serious games, image mosaicking, field of view, javascript, html, opensource

## Abstract

**Background:**

During keyhole surgery, the surgeon is required to perform highly demanding tasks while only being able to see part of the patient’s anatomy. This limited field of view is widely cited as a key limitation of the procedure, and many computational methods have been proposed to overcome it. However, the precise effects of a limited field of view on task performance remain unknown due to the lack of tools to study these effects effectively.

**Objective:**

This paper describes our work on developing a serious game with 2 objectives: (1) to create an engaging game that communicates some of the challenges of keyhole surgery, and (2) to test the effect of a limited field of view on task performance. The development of a serious game that can be played by a wide range of participants will enable us to gather quantitative data on the effects of the reduced field of view on task performance. These data can inform the future development of technologies to help surgeons reduce the impact of a limited field of view on clinical outcomes for patients. The game is open source and may be adapted and used by other researchers to study related problems.

**Methods:**

We implemented an open-source serious game in JavaScript, inspired by the surgical task of selectively cauterizing blood vessels during twin-to-twin transfusion surgery. During the game, the player is required to identify and cut the correct blood vessel under different fields of view and varying levels of vascular complexity. We conducted a quantitative analysis of task performance time under different conditions and a formative analysis of the game using participant questionnaires.

**Results:**

We recruited 25 players to test the game and recorded their task performance time, accuracy, and qualitative metrics. Reducing the field of view resulted in participants taking significantly longer (P<.001) to perform otherwise identical tasks (mean 6.4 seconds, 95% CI 5.0-7.8 seconds vs mean 13.6 seconds, 95% CI 10.3-16.9 seconds). Participants found the game engaging and agreed that it enhanced their understanding of the limited field of view during keyhole surgery.

**Conclusions:**

We recruited 25 players to test the game and recorded their task performance time, accuracy, and qualitative metrics. Reducing the field of view resulted in participants taking statistically significantly longer (16.4 vs 9.8 seconds; P=.05) to perform otherwise identical tasks. Participants found the game engaging and agreed that it enhanced their understanding of the limited field of view during keyhole surgery.

## Introduction

### Background

Keyhole surgery presents various advantages when compared with open surgery. The primary reason is that manipulation of abdominal tissue is reduced, resulting in less scarring, trauma, and hemorrhaging. This reduces the demand on health care services as patients require shorter hospital stays due to less postoperative pain [1-3]. Although keyhole surgery offers advantages, there are also limitations, such as a limited field of view, reduced depth perception, and increased procedure times.

Keyhole surgery is performed using endoscopes (and laparoscopes, a rigid endoscope used in abdominal procedures). Endoscopes consist of a long thin tube, with a camera and light source attached at the end. The surgeon is unable to see the anatomy directly but instead relies on video relayed from the endoscope camera [4]. This video presents a significantly reduced field of view in comparison to open surgery [5].

The monitor reduces depth perception of the operating scene as surgeons must map between the 2D image on the monitor and the 3D anatomical structure of the patient [6]. Visual misperceptions can occur from the loss of binocular vision due to a decrease in depth perception [7]. This can also be caused by surgeon fatigue as the laparoscopic setup is cerebrally intensive and increases the cognitive load of surgeons [8].

Modern endoscopes provide high spatial resolution, but at the expense of a limited field of view [9]. The surgeon’s ability to view the surgical scene is limited by the narrow monocular field of view of the endoscopic camera [10]. In contrast to the panoramic view during open surgery, the endoscope only images small areas of the surgical scene at once [11]. The camera has a fixed field of view, requiring the surgeon to maneuver the laparoscope to the target region [12]. The limited field of view during endoscope surgery has been widely cited as a limitation, and this will be the focus of this research project.

### Image Mosaicking

Image mosaicking is an established technique to construct a single image of the increased field of view by aligning various partially overlapped images of the same scene. Computational mosaicking can increase the field of view without compromising spatial resolution. Recently, this technique has been heavily researched, and its application is used in numerous industries, such as surveillance, satellite mapping, and agriculture. Mosaicking has also been used in endoscopic surgery to overcome the limited field of view and assists surgeons in manipulating the surgical scene and planning surgeries [13]. Daga et al [14] demonstrated the use of computational mosaicking for spatial orientation and anastomoses localization during endoscopic procedures. Computational mosaicking in endoscopic surgery remains challenging due to inhomogeneous lighting [15] and uncontrolled movement of the endoscope combined with geometric image distortion from the endoscope camera [16]. Because of these challenges, research to develop enhanced mosaicking algorithms is ongoing; however, there remains little understanding of the likely benefits of computational mosaicking in this field. A recent study has shown that experienced laparoscopic surgeons are proficient at “mentally mosaicking,” which is the ability to effectively translate the 2D visual information into the 3D anatomical context [17]. Our research is inspired by the question of how to best deploy computational mosaicking taking into account the user’s ability to do the same task mentally.

A key question that remains difficult to answer is “What precisely are the benefits of mosaicking or otherwise enlarging the field of view?.” To put it in statistical terms, what is the expected effect size for a given change in the field of view? Estimating the effect size is essential for any power calculation required in a study on a proposed technology to enlarge the field of view. As the study becomes more realistic and onerous (eg, an in vivo study requiring human volunteers and expert surgeons), it becomes essential that a realistic required sample size can be calculated before gaining ethical approval. One way to estimate the effect size would be to perform a study measuring task performance versus field of view; however, such a study would require a large number of participants. Recruiting sufficient surgeons to perform such a study would be difficult and time-consuming.

### User Studies With Nonexpert Users

Our work builds on the recent work of Yoo et al [18] who asked whether nonsurgical participants could stand in for surgical participants in user studies. They compared performance between participants with different levels of surgical training when interacting with a surgical augmented reality system (also see [19]). Comparing surgeons with different levels of experience with nonsurgeons, they found important differences in the performance of surgeons and nonsurgeons, but also similarities that can be used to inform system design, concluding that nonsurgical users could act as useful stand ins for surgical users, particularly in the early stages of device development. We wanted to see whether, by creating an abstract and fun representation of the mosaicking problem, we could lower the bar to recruitment, thus making a prerecruitment power study less important (ie, we can easily recruit many people, and there is no risk to the participants), so we can keep recruiting until we have enough data to show the statistical significance and calculate an effect size to inform future work. One way to achieve this may be through a serious game, which creates a simplified representation of the clinical problem, allowing us to recruit nonexpert users.

### Serious Games

Serious games have become increasingly prevalent for educational purposes, partly due to advancements in technology [20]. Serious games fulfill an additional role beyond pure entertainment [21,22]. Research has shown that incorporating intrinsic motivation into games, such as challenges and curiosity, substantially increases user motivation [5]. Creating immersive game environments can generate a deeper understanding by allowing users to test their problem-solving and decision-making skills within a safe environment [23]. Serious games can be personalized and designed to support the acquisition of knowledge and skill development, showing the need for these games to evaluate learning progress through player feedback [24]. Providing an interactive learning environment with instant visual feedback, such as a score, encourages more involvement and leads to a greater desire to complete the task at hand [25].

Serious games have previously been applied in the field of surgical training; for example, Underground is a serious game for the Nintendo Wii U platform, and the psychomotor skills required by users to complete the game objectives are closely related to the laparoscopic motor skills required by surgeons. Jalink et al [26] concluded that playing Underground increased laparoscopic skill development. A very important point when considering the use of games to represent a complex real-world procedure such as surgery is construct validity, that is, can it be shown that the skills used during the game correlate with performance during surgery. Construct validity for Underground was demonstrated by IJgosse et al [27], proving a link between in-game performance and surgical skills. Perhaps more interestingly, links between in-game performance and suturing skills have been shown for games with no apparent link to surgery [28].

Similarly, Ou et al [29] demonstrated that surgical trainees with previous gaming experience performed better in terms of laparoscopic simulation performance, compared with their nongaming counterparts. Surgical serious games must measure specific game metrics to quantify user performance [30]. The Kheiron Training System is a serious game designed to test basic psycho-motor skills required during laparoscopic surgery by utilizing real laparoscopic instruments. However, no studies have provided validation of this game as a training platform or obtained data to quantify the effect of the limited field of view in this game [23]. Although these studies show that serious games can be applied to skill development for surgical tasks, none of them attempt to answer questions on whether surgical technology development can be informed by users’ task performance when playing serious games. In this paper, we demonstrate the use of a serious game to generate quantitative data to inform the ongoing development of computational mosaicking.

The remaining sections of this paper describe the development and testing of the serious game we are developing. The game is designed to enable gathering quantitative data on the effect of reduced field of view that will be applicable to keyhole surgery. These data will be useful for estimating effect sizes (and hence statistical power) for follow-up studies requiring more clinically representative participants and equipment. Alongside this, we also aimed to make the game engaging and fun to play, accessible to users of different abilities, and able to test the skills (hand-eye coordination and mental mosaicking) of different users.

### Game Design and Implementation

The game has 2 aims: first, to study the effect of a reduced field of view on task performance, and second, to create a game for public engagement that communicates the challenges of keyhole surgery to a nontechnical audience and explores how image mosaicking may help address these challenges. These 2 aims are somewhat contradictory. For a strict study of a reduced field of view, a randomized-level structure coupled with a strictly defined training protocol would be ideal, to avoid comparison results being confounded with learning effects [31]. For a game aimed at public engagement, however, we want something that is easy to play from level 1 and engages the player with increased challenges at each level.

For this study, we decided to focus on the latter aim, so we use a set-level structure with increasing challenge at each level. This was to ensure the user was in a flow state by increasing the skill level required to successfully complete each game level [32]. To reduce the impact of learning effects, the levels used for comparison were placed at the end of the sequential-level structure. Table 1 summarizes the level structure, the objectives for each level, and the skills developed for each level.

**Table 1:** Level structure showing the game features and skills required in each level. The game gets more difficult with each level, while incrementally introducing 1 of 3 challenges (more vessels, more complex shapes, and limiting the field of view).

Level	Vessels	Vessels intertwined	Field of view	Skills tested
1	1	No	Full	Hand-eye coordination
2	2	No	Full	Hand-eye coordination and decision-making
3	1	No	Limited	Hand-eye coordination and image mosaicking
4	2	Intertwined	Full	Hand-eye coordination, visual perception, and decision-making
5	2	Intertwined	Limited	Hand-eye coordination, visual perception, decision-making, and image mosaicking
6	3	Intertwined	Full	Hand-eye coordination, visual perception, and decision-making
7	3	Intertwined	Limited	Hand-eye coordination, visual perception, decision-making, and image mosaicking

We identified 4 key skills that we wanted to address in the game. The first skill is hand-eye coordination, which is needed for all surgeries. Successful surgery requires that the surgeon is able to accurately cut in the intended location. In actual surgery, this is complicated by the need to use specialized tools. This is particularly difficult for keyhole surgery where the action of the tools is reversed. In a previous study [33] with nonexpert recruits, we observed that the mental load required to use laparoscopic tools can overwhelm any effect observed from changing the control variables. If the cutting mechanism is made too realistic, it is likely that we would not observe a change in performance with the field of view, as nonexpert users might find the game too difficult. Therefore, we decided that the hand-eye coordination skill would just require the use of a mouse to position the cursor over the vessel and press the mouse button to commence a cut. This element of the game remains the same across all levels.

The second skill is decision-making in the form of curve tracing [34] (ie, given the choice of 2 vessels, which one should be cut?). The user is required to visually inspect each path and work out which one connects the 2 black dots. Our game was inspired by surgery to treat twin-to-twin transfusion, as this is an area where image mosaicking has been proposed to improve performance [14] in keyhole surgery. The surgeon selectively cauterizes placental blood vessels to separate the blood supply to each twin. This requires careful identification of each vessel and its path. We created a simplified representation of this to create a curve-tracing game. Our representation of this is abstract to enable nonexpert users to play, but elements such as multiple vessels and intertwining are introduced during the game. All levels apart from 1 and 3 have multiple vessels and the player must decide which is the correct vessel to cut.

The third skill is visual perception. In most keyhole procedures, differentiating one structure from another can be challenging, as human anatomy does not consist of regular shapes in high-contrast colors. Therefore, we designed the game with a low-color contrast between the vessels and the background. In levels 4-7, we introduced multiple intertwined vessels without color contrast to make it more difficult to distinguish between them.

The final skill is image mosaicking. During keyhole surgery, it is not possible to see the whole surgical scene, a situation referred to as the limited field of view. A skilled keyhole surgeon must be able to mentally reconstruct the whole anatomical scene from a series of partial views created as they move the endoscope around. We introduce this skill at level 3 using a spotlight effect, so the player can only see part of the scene at once and must move the spotlight around with the mouse to mentally reconstruct the scene and identify the correct vessel.

The 4 skills are combined in different ways as the game progresses. To study the potential effects of a limited field of view, we created 2 pairs of levels (4<>5 and 6<>7) that are identical except for the field of view, allowing for a comparison of results between these levels.

### Game Implementation

**Figure 1:**
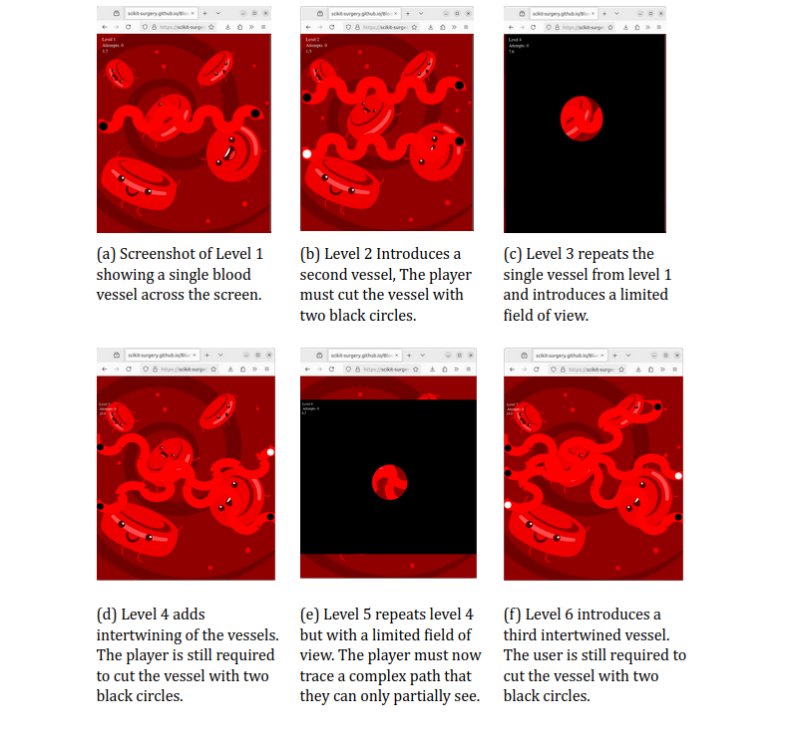
Examples of 6 game levels illustrating the incremental change in difficulty throughout the game. The full implementation of the game can be found at [35] or played directly at [36].

Figure 1 (also see [35,36]) shows screenshots of 6 game levels, illustrating the main game mechanics. Figure 1A shows the game at its most basic level with a single vessel on the screen. The user has identified the vessel and drawn a black line across the vessel to cut it. The time (in seconds) it took them to do this is displayed at the top left along with the number of attempts. A reduced field of view is introduced at level 4 (Figure 1C), with levels getting progressively harder until level 7 (Figure 1F), which has 3 intertwined blood vessels with a reduced field of view. Finally, level 7 has 3 intertwined blood vessels combined with a limited field of view.

The game was implemented in HTML and JavaScript and can be run in most modern web browsers. It is hosted as a static web page on GitHub. Allowing users to run the game directly from their browsers provides instant feedback, enhancing user interaction and engagement by displaying results immediately [33]. Game elements were created using the Phaser (version 3.60.0) [37] game framework. Phaser offers a configurable, open-source development library that supports small build sizes and fast loading times [38]. Additionally, it provides a wide range of tutorials and community support to facilitate development.

The game includes a timer to measure the time taken to complete each level but does not record results. Therefore, for the experiments, the results were recorded manually by the authors. For full technical details, the version of the game used in this paper, along with the data supporting the results, is archived on Zenodo [35].

### Vessel Creation and Cutting Logic

Vessels were represented as polygons, colored red, and stretched to fit the window width. Users navigated a pointer around the scene using their mouse. On levels with a reduced field of view, the mouse also moved the viewport (implemented using a circular bitmap mask), ensuring that the mouse pointer remained at the center of the viewport. Pressing and holding the mouse button started drawing a black line across the scene, while releasing the button ended the line drawing and incremented the attempt counter by 1. Upon completing the line drawing, a check determined whether the drawn line completely intersected the target vessel. If successful, a level completion message was displayed, and the level timer stopped. To prevent users from drawing a line across the entire scene—inevitably intersecting both sides of the polygon—a maximum line length of 45 units was enforced.

For levels with multiple blood vessels, circles were added to the end of each path, and users were required to cut only the blood vessel labeled with 2 black circles (see Figure 1C for an illustration).

Figure 1D demonstrates how the limited field of view was incorporated. Users could only view the game scene within the mask by moving their mouse across the screen. They had to navigate around the screen to locate the blood vessel marked with 2 black circles and use their mouse to cut it. The timer, displayed at the top of the game interface, stopped only after both sides of the correct blood vessel were fully intersected. If the user failed to completely intersect both sides of the blood vessel, the accuracy counter was incremented, and the timer continued. Users then had to try again by drawing a new line to cut the blood vessel.

The complete implementation of the game used in this publication is archived online [35]. The archive also includes links to newer development versions of the game and the URL provided to participants for accessing the game. This participant access URL leads to an index screen that contains links to the consent form, game instructions, and individual levels.

## Methods

### Study Design

We used the game to perform a single-arm user study with all users playing all levels of the game in the same order.

### Participant Recruitment

As discussed in the “Introduction” section, a key aim of the game design was to ensure that clinical experience was not required to play. Therefore, we deliberately avoided recruiting surgeons at this stage, although they were not excluded. The only inclusion criteria were age (participants had to be between 18 and 65 years old) and residency in the United Kingdom. Participants were recruited through the departmental email list (Medical Physics and Biomedical Engineering) or via a direct approach among the first author’s acquaintances.

Participant experiments were conducted either in person or via video calls and lasted approximately 15 minutes. Participants accessed the game through a URL [35].

Before playing the game, participants were asked, “What do you know about laparoscopic surgery?” and “Do you know the potential effects of a limited field of view in laparoscopic surgery?” to assess their prior knowledge of the domain. After completing a consent form, participants were provided with instructions on how to play the game.

Participants were instructed to play the game and complete each level in sequence, accessing each level through the game’s home page.

### Time to Complete Level

The in-game timer started automatically when participants clicked on a game level and stopped once they successfully cut the correct blood vessel. These data were recorded by the researcher for both in-person and remote experiments.

### Accuracy

A counter variable recorded the number of attempts each participant needed to successfully complete each level, increasing with each mouse click used to draw a new line.

### Participant Questionnaires

In addition to the pregame questionnaires, participants completed a postgame questionnaire, a NASA (National Aeronautics and Space Administration) Task Load Index [39] questionnaire, and a System Usability Scale [40] questionnaire after finishing all 7 levels. Full details of the questions can be found in the “Results” section.

### Ethical Approval

This study was approved by University College London’s Research Ethics Committee (reference number 24249_001). Informed consent was obtained from all individual participants included in the study.

## Results

### Participant Recruitment

We recruited 25 participants from a range of backgrounds, experiences, and ages for this study. The recruitment approach led to a high number of master’s students with advanced knowledge of laparoscopic surgery and computer science, as well as students specializing in other sciences. Additionally, working professionals from various industries were recruited, some with little to no understanding of laparoscopic surgery. One participant had medical experience and reported knowledge of keyhole surgery. This diverse participant pool was selected to gather a broad range of responses and perspectives.

We did not record details of participants’ prior gaming experience or their computer usage. However, as some participants were known to the authors, we can anecdotally state that those who used computers less tended to complete tasks more slowly and found the user interface harder to navigate. The participants with medical experience did not appear to perform differently from the main population.

### Time to Complete Level

The mean and SD of the time to complete each level are shown on the left-hand side of Table 2, along with 95% CIs for the mean.

**Table 2:** Mean (SD) and 95% CI for time and number of attempts to complete each level.

Level	Participant time results (seconds)			Participant accuracy (attempts)	
	Mean (SD)	95% CI	Mean (SD)		95% CI
1	5.7 (3.6)	4.2-6.9	1.3 (2.0)		0.5-2.1
2	3.3 (2.6)	2.2-4.4	1.2 (0.7)		0.9-1.5
3	7.2 (2.8)	6.0-8.4	1.4 (1.1)		0.9-1.9
4	6.4 (3.3)	5.0-7.8	1.6 (1.1)		1.2-2.0
5	12.5 (5.7)	10.2-14.8	1.4 (0.7)		1.1-1.7
6	9.8 (4.8)	7.8-11.8	1.8 (0.9)		1.4-2.2
7	16.4 (11.0)	11.9-20.9	1.6 (1.2)		1.1-2.1

**Figure 2:**
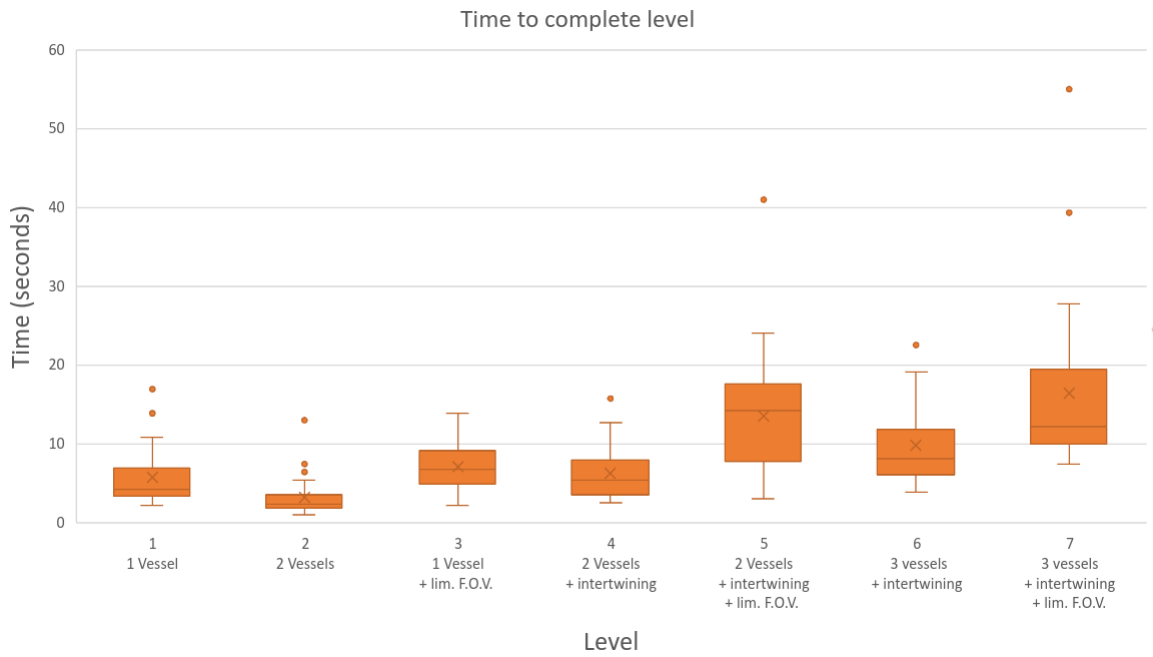
Box plot displaying the average time to complete each level. F.O.V.: field of view.

Figure 2 presents a boxplot of level completion times alongside a brief description of each level’s features. The figure shows a general trend of increasing completion time from left to right, corresponding with an increasing level of complexity. On average, level 2 was completed the fastest (in 3.3 seconds). This level featured 2 blood vessels displayed on the game interface without a restricted field of view. By contrast, level 7—the most challenging level—took the longest time to complete, with an average time of 16.4 seconds. This level featured 3 intertwined blood vessels following a complex path and was constrained by a limited field of view.

While the general trend in Figure 2 shows increasing completion times as level complexity increases, there are 2 exceptions when the field of view is restricted. Level 4 (with 2 blood vessels) had a shorter completion time than level 3 (which featured a single blood vessel and a limited field of view). Similarly, level 6 (with 3 intertwined blood vessels) was completed faster than level 5 (which had 2 intertwined blood vessels and a limited field of view). This suggests that a restricted field of view has a greater impact on performance than on other factors examined in this study. The effect of limiting the field of view can be estimated by comparing results from levels that were identical except for this restriction (ie, comparing level 4 with level 5 and level 6 with level 7).

We compared the completion times for levels 4 and 5 using a Welch 2-sample *t* test, which yielded a *P* value of <.001 indicating a statistically significant impact of limiting the field of view. The Cohen *d* effect size was 1.19, suggesting a large effect. A similar comparison between levels 6 and 7 produced a *P* value of .009 and an effect size of 0.79, further supporting the significant impact of a restricted field of view.

### Accuracy

The number of attempts is displayed on the right-hand side of Table 2. A Welch 2-sample *t* test confirmed no significant differences in the number of attempts across levels (the minimum *P* value was .23 between levels 5 and 6). The numerical results and analysis scripts referenced above are archived along with the game code in [35].

### Participant Questionnaires

To assess participants’ prior knowledge, all participants were asked, “What do you know about laparoscopic surgery?” and “Do you know the potential effects of a limited field of view in laparoscopic surgery?” before playing the game. All 25 participants recognized that laparoscopy is a type of surgical procedure; however, their level of understanding varied significantly depending on their occupation and personal experiences. Participants from the researcher’s student cohort were highly knowledgeable about this topic and understood both the advantages and limitations of this minimally invasive procedure. By contrast, participants from a nonmedical background had limited awareness of the benefits of laparoscopic surgery and the potential impact of a restricted field of view.

The results of the postgame questionnaire are shown in Table 3. The questionnaire was completed by 22 participants. Three participants commented on the game’s background color, suggesting that a greater contrast between the background and blood vessels would improve visibility. Four participants stated that they enjoyed the game timer, as it heightened their competitiveness under time pressure. Two participants mentioned feeling frustrated due to their limited experience with a Mac laptop and its built-in mouse, which hindered their ability to complete levels quickly.

**Table 3:** Participant questionnaire results: percentage and absolute number of participants who answered yes when answering the questionnaire after completing the game.

Question	Yes, n/N (%)
Did playing this game enhance your understanding of the limited field of view in laparoscopic surgery?	20/22 (91)
Do you think this game is clinically relevant?	19/22 (86)
Do you think the difficulty increased with each level?	20/22 (91)
Did you find the game engaging?	22/22 (100)
Did you find the game layout visually pleasing?	22/22 (100)

Participants also completed a NASA Task Load Index [39] questionnaire, rating each workload demand on a scale of 1-10. For the questions related to demand (1-3, 5-6) a score of 1 is described as “very low” and a score of 10 as “very high.” For question 4, a score of 1 is “perfect” and 10 is “failure.” Table 4 presents the average scores for the 6 workload demands. The results indicate that participants felt neutral about the mental demands required to complete the tasks in this serious game. Physical demand received the lowest rating, with an average score of 1.6, while performance workload was rated the highest. Effort workload scores varied significantly, with widely dispersed data resulting in a neutral average of 5.3. Participants rated their frustration levels relatively low, with an average score of 2.8.

Additionally, participants completed a System Usability Scale questionnaire [40], the results of which are shown in Table 5.

**Table 4:** NASAa Task Load Index results: the average score out of 10 for each demand.

NASA Task Load Index	Mean score
Mental: How mentally demanding was the task?	4.9
Physical: How physically demanding was the task?	1.6
Temporal: How hurried or rushed was the pace of the task?	5.6
Performance: How successful were you in accomplishing what you were asked to do?	7.7
Effort: How hard did you have to work to accomplish your level of performance?	5.3
Frustration: How insecure, discouraged, irritated, stressed, and annoyed were you?	2.8

^a^NASA: National Aeronautics and Space Administration.

**Table 5:** System Usability results: the average score out of 5 for each demand in the System Usability Scale.

System Usability Scale	Mean score
I think that I would like to use this system frequently	3.2
I found the system unnecessarily complex	1.3
I thought the system was easy to use	4.3
I think that I would need the support of a technical person to be able to use this system	1.5
I found that the various functions in this system were well integrated	3.8
I thought there was too much inconsistency in this system	1.7
I would imagine that most people would learn to use this system very quickly	4.6
I found the system very cumbersome to use	1.6
I felt very confident using the system	4.3
I needed to learn a lot of things before I could get going with this system	1.2

## Discussion

### Principal Findings

The qualitative results from participant questionnaires suggest that we have successfully created an engaging game that can facilitate discussions about the challenges of keyhole surgery with nontechnical audiences. The quantitative results, based on comparisons of level completion times, indicate that our game can provide valuable insights into the effects of a limited field of view on task performance.

### Game Design

Our game design was a balance between creating an engaging experience by gradually increasing the difficulty of each level and enabling a paired comparison between different conditions. Our results indicate that we largely succeeded in both objectives. As shown in Table 3, all participants found the game engaging, and we observed a statistically significant difference in performance when the field of view was reduced (see Figure 2).

Randomizing the level structure might have resulted in a more robust test of our hypotheses but would likely have come at the expense of participant engagement. Maintaining a continuous flow state throughout the game was crucial, as the flow has a positive impact on learning and is strongly linked to user attention and focus. Research suggests that when a user’s attention is directed toward a limited set of stimuli, irrelevant perceptions and distractions are minimized [41]. Additionally, studies have demonstrated that sensory and cognitive curiosity increases when users find a game intrinsically interesting, thereby enhancing engagement [42,43]. Notably, all 22 participants who completed the questionnaire agreed that this serious game was engaging.

Three participants commented on the color contrast between the game background and blood vessels, suggesting that a greater difference in color would have helped them distinguish between the 2, especially in the harder levels. The low contrast between the background and blood vessels was an intentional design choice, as the illumination of the surgical scene is limited during laparoscopic surgery [15].

Additionally, bodily fluids, such as blood, can further obscure the surgeon’s view of the anatomical context. The game design aimed to replicate these challenges by maintaining low contrast, requiring players to focus and use precision to identify and cut the correct blood vessels. Future work could explore fine-tuning the display for different applications of the game.

### Learning the Anatomy

There is evidence of a learning effect between levels 1 and 2, as level 2 was completed faster on average and with fewer attempts than level 1 (see Table 2). Observations of participants attempting level 1 indicated that they were initially unaware of the maximum line length they could draw with their mouse. It is likely that the lower average number of attempts in level 2 resulted from participants becoming aware of this game mechanic.

Levels with a more complex vessel pattern (levels 4 and 6) required more attempts than those where the pattern remained the same or had no intertwining (levels 2, 3, 5, and 7); however, these differences were not statistically significant. The finding that levels with a reduced field of view took longer to complete but did not require more attempts suggests that the mechanisms for mental mosaicking and learning the anatomy may be distinct processes.

### Accessibility

This game was designed to be accessible to users of all abilities. The participant pool was diverse, with varying levels of knowledge and experience in laparoscopic surgery and web-based games. Participants ranged in age from 18 to 57 years, with 2 reporting dyslexia and 1 reporting dyspraxia. All 25 participants successfully completed all levels, suggesting that the game was accessible to them. However, no participants had color blindness or vision deficiencies. Given the low contrast between the game background and blood vessels and the fact that the correct blood vessels were identifiable only by black circles, it cannot be concluded that the game is accessible to users with visual impairments. Results from the NASA Task Load Index indicated varied effort workload scores, suggesting that some participants had limited experience using a laptop. As a result, the game may be less accessible to players with no prior laptop or gaming experience.

### Limitations and Future Work

At present, the game presents a very simplified representation of the surgical environment. This simplification limits the game’s direct relevance to surgery. The simplification was a deliberate decision to keep the game accessible to the widest possible user base; however, future work may require a more clinically realistic environment. Repeating the experiment with a sample of surgeons, controlling for specialty and expertise, will help validate our methodology of recruiting nonexpert participants. Bearing in mind the results of Yoo et al [18], we still expect to see a correlation between the field of view and task completion time; however, we would not expect the results to be identical. Furthermore, the results would vary between levels of surgical experience and specialty. Surgeons would bring differing levels of prior knowledge that would alter game performance. Comparing the results between different user groups might yield useful information about the differences between trained surgeons and the general population.

The game’s simplicity makes it impossible at present to fully understand the impact of learning effect on the results. The fact that the level pairs we used to compare between different field of view settings (4<>5 and 6<>7) were otherwise identical means that our results may underestimate the impact of reducing the field of view due to the participants learning from the preceding level. Future work could look at introducing more complex level progression to control for this.

To increase the clinical relevance of the game, the graphics and design could be changed to represent a more clinical environment. For example, it would be relatively easy to change the backdrop to a screenshot taken from a clinical procedure with vessels overlaid in more clinically realistic colors. Artifacts such as smoke and bleeding often seen in keyhole surgery could also be added, but would require significantly more programming work. It would then make interesting future work to compare the performance on this more realistic game between surgeons and nonsurgeons. We are also exploring ways to incorporate a negative scoring system to penalize mistakes, such as cutting the wrong blood vessel, and enable better analysis of how the field of view affects the errors made.

To investigate the impact of computational image mosaicking on task performance, additional levels will be required. At a basic level, users could use their mouse to “paint” on the scene, revealing the blood vessels underneath. This would create a larger field of view, simulating computational image mosaicking. As the game becomes more complicated, it is likely that the effect sizes will decrease, requiring a larger sample size to demonstrate statistical significance. A key advantage of our approach is its ability to support the recruitment of large numbers of participants.

Work is ongoing to improve scene management, making it easier for the user to move from one level to the next. Efforts are also underway to gather results using an automated database backend.

### Conclusions

Our serious Blood Vessel Game was used to demonstrate a quantifiable effect of a limited field of view on task performance time, with the same task taking between 60% and 100% longer when the view was restricted. No effect on task accuracy was detected. Our results represent the first time this effect has been quantified in this way. The game also serves as an engaging educational tool for discussing the impact of a limited field of view on task performance, with 20 out of 22 (91%) participants agreeing that the game was educational and all (22/22, 100%) agreeing that it was engaging. The game is entirely open source, and we welcome contributions to enhance its usefulness.

## Abbreviations

NASA: National Aeronautics and Space Administration

## Data Availability

The game implementation and data used in this paper are freely available at [35].
